# Mice Doubly-Deficient in Lysosomal Hexosaminidase A and Neuraminidase 4 Show Epileptic Crises and Rapid Neuronal Loss

**DOI:** 10.1371/journal.pgen.1001118

**Published:** 2010-09-16

**Authors:** Volkan Seyrantepe, Pablo Lema, Aurore Caqueret, Larbi Dridi, Samar Bel Hadj, Stephane Carpentier, Francine Boucher, Thierry Levade, Lionel Carmant, Roy A. Gravel, Edith Hamel, Pascal Vachon, Graziella Di Cristo, Jacques L. Michaud, Carlos R. Morales, Alexey V. Pshezhetsky

**Affiliations:** 1 Division of Medical Genetics, Centre Hospitalière Universitaire Sainte-Justine, University of Montréal, Montréal, Quebec, Canada; 2 Neurology, Centre Hospitalière Universitaire Sainte-Justine, University of Montréal, Montréal, Quebec, Canada; 3 INSERM U858 and Laboratoire de Biochimie “Maladies Métaboliques”, Centre Hospitalière Universitaire Toulouse, Toulouse, France; 4 Department of Biochemistry & Molecular Biology, University of Calgary, Calgary, Alberta, Canada; 5 Montreal Neurological Institute, McGill University, Montréal, Quebec, Canada; 6 Department of Veterinary Biomedicine, Faculty of Veterinary Medicine, University of Montreal, Montreal, Quebec, Canada; 7 Department of Anatomy and Cell Biology, Faculty of Medicine, McGill University, Montréal, Quebec, Canada; The Jackson Laboratory, United States of America

## Abstract

Tay-Sachs disease is a severe lysosomal disorder caused by mutations in the *HexA* gene coding for the α-subunit of lysosomal β-hexosaminidase A, which converts G_M2_ to G_M3_ ganglioside. *Hexa^−/−^* mice, depleted of β-hexosaminidase A, remain asymptomatic to 1 year of age, because they catabolise G_M2_ ganglioside via a lysosomal sialidase into glycolipid G_A2_, which is further processed by β-hexosaminidase B to lactosyl-ceramide, thereby bypassing the β-hexosaminidase A defect. Since this bypass is not effective in humans, infantile Tay-Sachs disease is fatal in the first years of life. Previously, we identified a novel ganglioside metabolizing sialidase, Neu4, abundantly expressed in mouse brain neurons. Now we demonstrate that mice with targeted disruption of both *Neu4* and *Hexa* genes (*Neu4* ^−/−^;*Hexa* ^−/−^) show epileptic seizures with 40% penetrance correlating with polyspike discharges on the cortical electrodes of the electroencephalogram. Single knockout *Hexa* ^−/−^ or *Neu4* ^−/−^ siblings do not show such symptoms. Further, double-knockout but not single-knockout mice have multiple degenerating neurons in the cortex and hippocampus and multiple layers of cortical neurons accumulating G_M2_ ganglioside. Together, our data suggest that the Neu4 block exacerbates the disease in *Hexa^−/−^* mice, indicating that *Neu4* is a modifier gene in the mouse model of Tay-Sachs disease, reducing the disease severity through the metabolic bypass. However, while disease severity in the double mutant is increased, it is not profound suggesting that Neu4 is not the only sialidase contributing to the metabolic bypass in *Hexa* ^−/−^ mice.

## Author Summary

Tay-Sachs disease is the second most common lysosomal storage disorder, especially frequent in Ashkenazi Jews and French Canadians. The disorder is caused by mutations in the gene coding for lysosomal β-hexosaminidase A (*HexA*), resulting in accumulation of G_M2_ ganglioside in neurons followed by progressive neurologic degeneration, fatal in early childhood. However mice, depleted of HexA, remain asymptomatic to at least 1 year of age, owing to the ability of these mice to catabolise stored G_M2_ ganglioside via a lysosomal neuraminidase into glycolipid G_A2_ further processed by β-hexosaminidase B, thereby completely bypassing the *HexA* defect. Our current study provides an explanation why the disease is severe in humans but not in mice. We showed that mice depleted of both *HexA* and ganglioside neuraminidase 4 (*Neu4*) show epileptic seizures similar to that often observed in Tay-Sachs patients. Single *HexA* or *Neu4* knockout mice do not show such symptoms. Further, double-knockout but not single-knockout mice have multiple degenerating cortical and hippocampal neurons and multiple layers of cortical neurons accumulating G_M2_ ganglioside. Our data suggest that the *Neu4* depletion exacerbates the disease in *HexA* knockout mice, supporting the view that *Neu4* is one of the modifier genes in the mouse model of Tay-Sachs disease.

## Introduction

Tay-Sachs disease (reviewed in [1]) is the second most common lysosomal storage disorder [2], especially frequent in two populations: Ashkenazi Jews (carrier frequency 3.4%) [3] and French Canadians from Gaspé-Bas St-Laurent region of Quebec (carrier frequency 5–7%) [4]. The disorder is caused by mutations in the *HexA* gene coding for the α-subunit of lysosomal β-hexosaminidase A (HexA), which removes N-acetyl-glucosamine residue from G_M2_ ganglioside, converting it to G_M3_ ganglioside. This causes accumulation of G_M2_ ganglioside in neurons of affected patients with subsequent neuronal death, resulting in progressive neurologic degeneration. Classic Tay-Sachs disease is characterized by onset of muscle weakness and hypotonia in infancy associated with myoclonic jerking upon auditory stimulation, followed by spasticity, dementia, blindness and epilepsy, with death in the second to fourth year of life [1]. Less frequent juvenile and adult forms of the disease are characterized by later onset and milder symptoms [1]. The clinically similar disorder, Sandhoff disease is caused by the mutations in the *HexB* gene coding for the β-subunit of hexosaminidase A which results in simultaneous deficiency of Hex A and HexB [1].

Important insight into disease mechanism and the development of therapies for Tay-Sachs disease have come from studying the mouse model for the disorder, genetically targeted mice with a disrupted *Hexa* gene. Independent publications from several laboratories [5]–[7] reported that disruption of the *Hexa* gene in mouse embryonic stem cells resulted in mice that showed no neurologic abnormalities to one year of age, although they exhibited biochemical and pathologic features of the disease [8]. In contrast, mice in which the *Hexb* gene was disrupted (a model of human Sandhoff disease) were severely affected by 2–3 months of age and died 4–6 weeks later [5]–[6]. The phenotypic differences between the two mouse models were explained by a major difference in the ganglioside degradation pathways in humans and mice. In particular, it was reported [5]–[6] that mouse neurons are enriched in a lysosomal ganglioside sialidase activity that removes the terminal sialic acid from G_M2_ ganglioside converting it into glycolipid G_A2_ which is further degraded by HexB. Most recent study in embryonic and postnatal brains and cultured neural cells derived from Tay-Sachs and Sandhoff mouse models shows that alternative roots for the formation of G_M3_ ganglioside also exist in *Hexb^−/−^* cells but they do not sufficiently reduce G_M2_ storage [9].

Recent studies from our laboratory suggested that lysosomal sialidase/neuraminidase 4 (Neu4) may function as the ganglioside sialidase acting in Hexa^−/−^ mice [10]. Neu4 previously cloned by us [11] and other groups [12]–[14] is ubiquitously expressed in human tissues and is active against all types of sialylated glycoconjugates including oligosaccharides, glycoproteins and gangliosides [11]–[14]. Our data showed that Neu4 in the presence of detergents or lysosomal activator proteins actively desialylated G_M2_ ganglioside [10]. In contrast, another lysosomal sialidase, neuraminidase 1 (Neu1) had very little activity towards gangliosides [10]. Genetically-targeted mice with knock-out of the *Neu4* gene had lysosomal storage bodies in lung macrophages, spleen macrophages and lymphocytes and showed increased levels of gangliosides, ceramide, cholesterol and fatty acids in brain, liver, lungs, and spleen [10]. Finally, we showed that transfection of cultured neuroglia cells from a Tay-Sachs patient with a Neu4-expressing plasmid restored normal morphological phenotype of the cells and corrected the impaired metabolism of G_M2_ ganglioside via glycolipid G_A2_, thereby acting as a bypass for the HexA deficiency [10].

In the current work, we assessed whether Neu4 is the enzyme responsible *in vivo* for the metabolic bypass of the HexA defect in the mouse model of Tay-Sachs disease by studying mice with a double deficiency of Neu4 and HexA.

## Results

### Generation of HexA/Neu4-deficient mice

Mice with a combined deficiency of Neu4 sialidase and HexA were obtained by intercrossing the previously described *Neu4* and *Hexa* knockout mouse models, both in C57BL/6NCrl genetic backgrounds. Doubly homozygous *Neu4* ^−/−^;*Hexa* ^−/−^ progeny were viable and their genotypes were confirmed by PCR of tail DNA (Figure S1). The absence of *Neu4* transcripts in total mRNA extracted from the brain of *Neu4^−/−^;Hexa^−/−^* mice was confirmed by RT-PCR (Figure S2). HexA activity assayed using the HexA-specific substrate, 4MU-β-D-N-acetylglucosamine sulfate, confirmed its complete deficiency (Figure S3). Acid sialidase activity, assayed using 4MU-NeuAc as substrate, was significantly reduced to ∼30% activity, as anticipated (data not shown). Partial reduction of acid sialidase activity reflects the presence of other sialidases that contribute to the net sialidase activity against 4MU-NeuAc [10]. *Neu4^−/−^;Hexa^−/−^* mice were born in the frequency expected from Mendelian inheritance, indicating that simultaneous disruption of the *Hexa* and *Neu4* genes does not cause embryonic lethality. Up to the age of 3 months, *Neu4^−/−^;Hexa^−/−^* mice showed normal growth and were behaviorally and physiologically indistinguishable from wild type animals. Both males and females were fertile and could be bred to produce knockout litters.

### Seizure activity

Starting from 3 months of age *Neu4^−/−^;Hexa^−/−^* mice began to show episodes of seizures provoked by handling of animals and loud sounds (Video S1). The symptoms were observed in ∼40% of double-deficient animals (6 out of 14) but never in wild type mice or in mice homozygous for the *Neu4* or *Hexa* mutant alleles. One mouse developed paralysis on the right hind limb (Figure S4).

**Figure 1:**
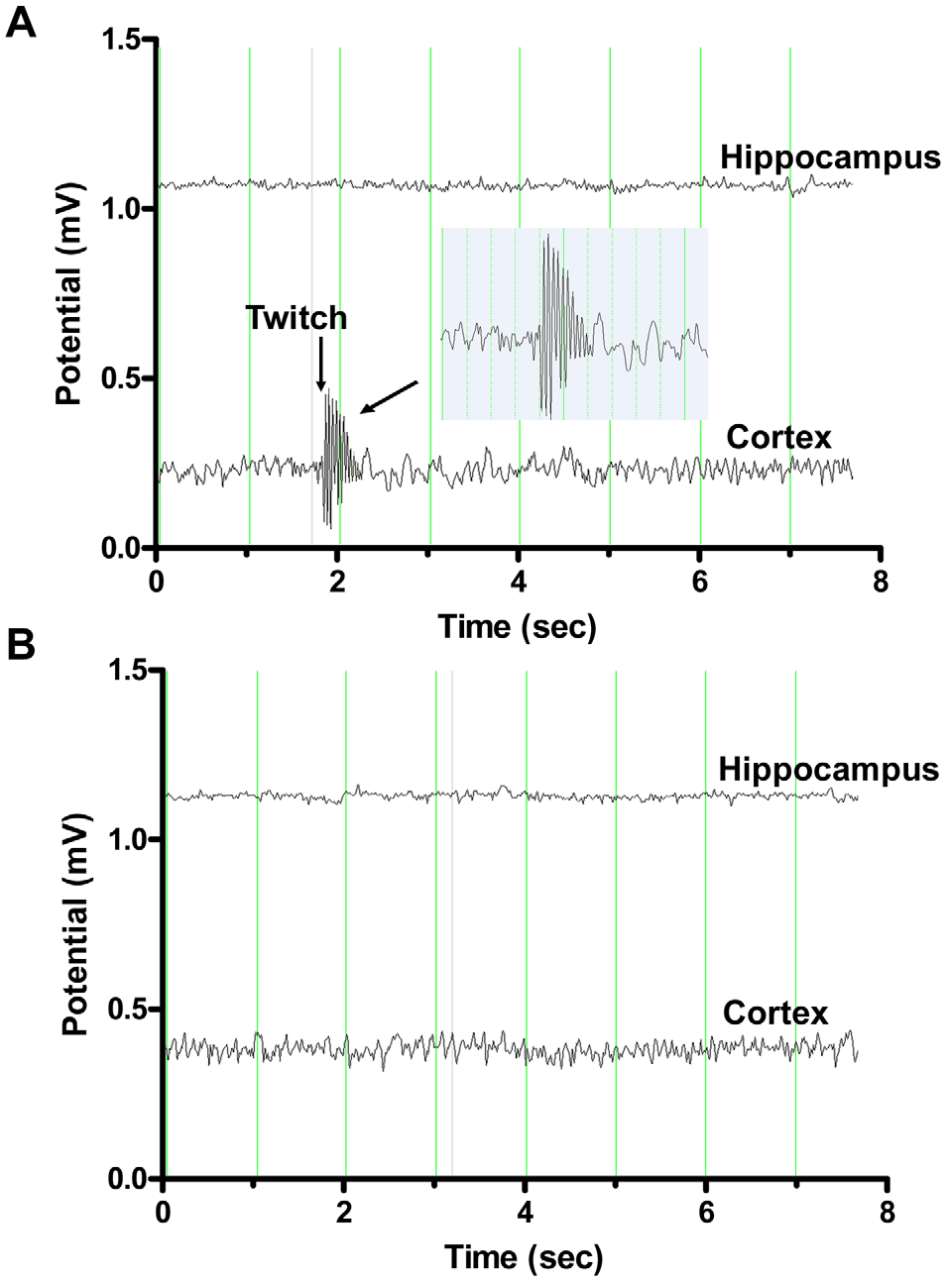
Representative electroencephalography (EEG) recordings from double deficient *Neu4^−/−^;Hexa^−/−^* and single-deficient *Hexa* ^−/−^ mice. EEG of *Neu4^−/−^;Hexa^−/−^* mouse (**A**) shows simultaneously with a myoclonic jerk (indicated as “twitch” on the figure) high amplitude polyspike discharges involving the cortical electrode with no diffusion to the underlying hippocampus. The EEG of *Hexa* ^−/−^ mouse (**B**) is normal. Inset shows the enlargement of the myoclonic event. Each vertical line on the graph is 1 sec, voltage scale is 30 µV/mm.

Six animals, three *Neu4^−/−^;Hexa^−/−^* double mutants and three *Neu4^+/+^;Hexa^−/−^* single mutants, were implanted for video-electroencephalogram (EEG) monitoring at the age of 16 weeks. All three *Neu4^−/−^;Hexa^−/−^* mice exhibited generalized epileptic seizures characterized clinically by myoclonic jerks. The EEG during the seizures demonstrated polyspike discharges that always involved the cortical contact (Figure 1A) and sometimes the hippocampal contacts (not shown). Occasionally, discharges were observed with no clinical accompaniment. EEG of all three *Neu4^+/+^;Hexa^−/−^*mice were normal (Figure 1B).

### Motor activity, behavioral changes and learning ability

At the age of 4 months, double mutants, single mutants and wild-type mice performed equally well in the rotarod test, indicating the absence of obvious motor deficits in mice with a loss of *Neu4* and *Hexa* (Figure S5). However, neurological examination demonstrated that *Neu4^−/−^;Hexa^−/−^* mice showed tremor during locomotion, wide based stance, and increased spasticity in anterior and posterior limbs when held by the tail. These abnormalities were not seen in wild type or single knockout animals. At 4 months of age, all animals showed normal grid walking. One month later, the *Neu4^−/−^;Hexa^−/−^* mice, but not the single knockouts, demonstrated weakness on grid walking and weakness on force test when placed on side of the cage.

The open field test was used at 4 months to measure behavioral responses through exploratory behaviors which are altered by the level of anxiety [15]. Rats and mice tend to avoid brightly illuminated, novel, open spaces, so the open field environment acts as an anxiogenic stimulus and allows the measurement of anxiety-induced locomotor activity and exploratory behaviors. Although at the first evaluation, double mutants tended to explore more than the single mutants, this did not reach statistical significance. More importantly, both groups showed a similar travel distance and adaptation rate when compared at day 5 (Figure S6).

Finally to rule out any hippocampal dysfunction associated with the spontaneous seizures, we performed the Morris Water Maze test, a visuo-spatial task shown to be abnormal in animals with spontaneous limbic seizures. Both *Hexa^−/−^* and *Neu4^−/−^; Hexa^−/−^* mice at 4 months had no visual or motor deficit since they reached the platform as rapidly as wild-type mice in the visible platform pre-training test (not shown). All groups showed a comparable learning curve with similar average escape latency to reach the hidden platform (Days 4–8, Figure S7). When tested for memory in the probe trial on day 8, both groups performed similarly to wild type, although there was a tendency for the *Neu4^−/−^;Hexa^−/−^* mice to spend less time and perform fewer passes in the target quadrant (Figure S8).

### Brain pathology

Microscopic examinations were performed on coronal and sagittal paraffin-embedded sections of brain tissues. The study was conducted in the following experimental groups: *Neu4^−/−^;Hexa^−/−^* mice, either showing or not showing epileptic crises; their littermates with single deficiency of Neu4 (*Neu4^−/−^;Hexa^+/+^*) or Hex A (*Neu4^+/+^;Hexa^−/−^*); and wild type littermates used as controls. For comparative purpose the study was conducted on mice at the age of 6 months. The paraffin sections (5 µm thick) were stained with hematoxylin and eosin and viewed by light microscopy. The examination of the tissues showed a normal organization of the different cortical layers and hippocampal neurons in all groups (not shown). At higher magnification a large number of cortical neurons showed a vacuolated cytoplasm in the *Neu4^−/−^;Hexa^−/−^* mice, either with or without seizures, as well as in their littermates with deficiency of Hex A only (*Neu4^+/+^;Hexa^−/−^*). The brain sections of *Neu4^−/−^;Hexa^+/+^* and wild type mice did not contain vacuolated neurons (not shown).

**Figure 2:**
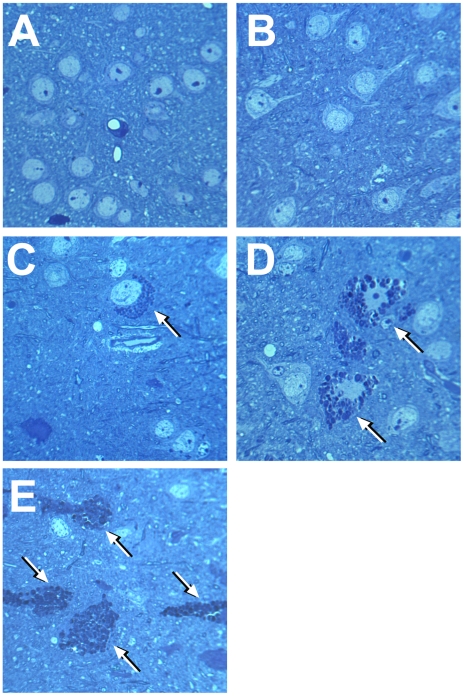
Light microscopy of neurons in deep cortex of wild type mice, single *Neu4^−/−^* or *Hexa^−/−^* knockouts and in double *Neu4^−/−^;Hexa^−/−^* knockouts without and with seizures. Affected neurons containing vacuolated cytoplasm shown by arrows are present in *Hexa^−/−^* knockouts (**C**) and in double knockouts without (**D**) and with (**E**) seizures, but absent in wild type mice (**A**) and single *Neu4^−/−^* knockouts (**B**). Magnification ×1000. Panels represent typical images obtained for 3 mice for each genotype.

**Figure 3:**
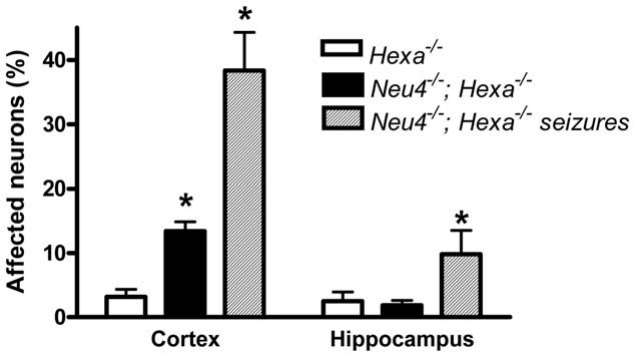
Percent of affected neurons in cortex and hippocampus. Normal and affected (vacuolized) neurons were counted in 9 epon sections from similar cortical and hippocampal regions of each mouse; 3 mice were studied for each genotype. * - significantly different from *Hexa* ^−/−^.

To study in detail the vacuolated neurons, we examined coronal sections through the mid-region of brains obtained from *Neu4^−/−^;Hexa^−/−^* mice, with or without seizures, as well as from their wild type littermates and those with single deficiencies. Both cortex and hippocampus were further trimmed into small (∼1 mm^3^) cubes and embedded in epon for pathological examination. One µm thick sections of the brain tissues were cut with an ultramicrotome and stained with toluidine blue. In all brain epon sections of wild type mice, the cortical neurons were normal (Figure 2A). In *Neu4* deficient mice (*Neu4^−/−^;Hexa^+/+^*), cortical neurons were mostly normal except for few dark degenerating neurons in deep layers of the cortex, possibly corresponding to pyramidal cells (Figure 2B and Figure S9C). In contrast, brains of mice with HexA deficiency only (*Neu4^+/+^;Hexa^−/−^*) contained more degenerating neurons in the same deep cortical layers (Figure 2C and Figure S9E). The superficial neurons of the cortex were rarely affected. Brains of double knockout (*Neu4^−/−^;Hexa^−/−^*) mice without seizures also showed a deep cortical layer of affected neurons but with higher frequency then in the *Neu4^+/+^;Hexa^−/−^* mice (Figure 2C and Figure S9G). Double knockout mice with seizures showed multiple cortical layers with even larger number of affected neurons (Figure 2D and Figure S9I). High power images of affected neurons showed that they contained an extensive accumulation of cytoplasmic vacuoles stained by toluidine blue (Figure 2). Hippocampal neurons exhibited a normal morphology in the brains of the wild type mice, in mice with single *Neu4^+/+^;Hexa^−/−^* and *Neu4^−/−^;Hexa^+/+^* deficiencies, as well as in mice with double deficiency not showing seizures, whereas hippocampal areas of mice with double deficiency and seizures contained numerous neurons with an extensive accumulation of cytoplasmic vacuoles stained by toluidine blue (Figure S9). In order to quantify these pathological changes, normal and affected neurons were counted in 6–9 epon sections from similar randomly chosen cortical and hippocampal regions of each mouse. The results showed that the number of affected cortical and hippocampal neurons in the mice with double deficiency of Neu4 and HexA (*Neu4^−/−^;Hexa^−/−^*) with seizures was significantly increased as compared to both single HexA deficient (*Neu4^+/+^;Hexa^−/−^*) mice and double deficient mice not showing seizures (Figure 3).

**Figure 4:**
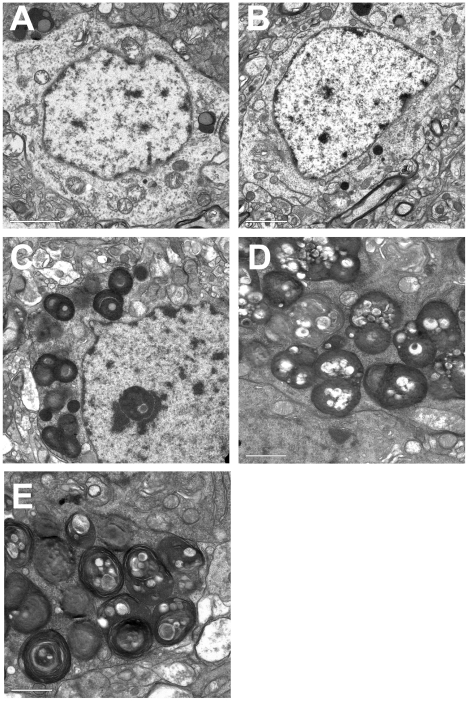
Electron micrographs of mouse brain tissues. Cortical neurons from wild type (**A**) and *Neu4^−/−^* mice (**B**) show normal lysosomes. Cortical neurons from *Hexa^−/−^* (**C**) knockouts, and double (*Neu4^−/−^;Hexa^−/−^*) knockouts without (**D**) and with seizures (**E**) show accumulation of whorls of membranes in lysosomes characteristic of neurons of Tay-Sachs patients. Bars range between 1 and 2 µm. Panels represent typical images obtained for 3 mice for each genotype.

To further study the morphology of affected neurons ultrathin sections from the mapped trimmed blocks containing the affected neurons were stained with lead citrate and uranyl acetate and observed under the electron microscope. For control purpose, similar regions were mapped in wild type brains. High power images showed that while normal neurons contained electron dense lysosomes, the affected neurons contained large irregular lysosomes presenting an accumulation of whorls of membranes typical of lysosomal storage defects (Figure 4 and Figure S10).

### Accumulation of G_M2_ ganglioside in the brain of *Neu4^−/−^;Hexa^−/−^* mice

**Figure 5:**
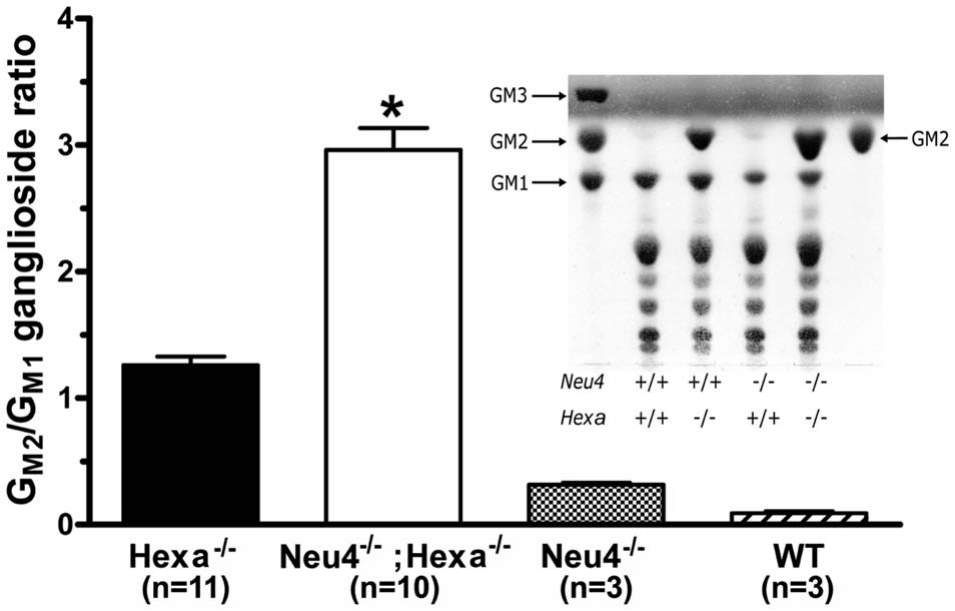
Alteration of G_M2_ ganglioside catabolism in the brain tissues of *Neu4^−/−^*;*Hexa^−/−^* mice. Histograms show the ratio of G_M2_ and G_M1_ gangliosides as measured by TLC in the extracts of brain tissues from wild-type (*WT*); *Neu4^−/−^*,*Hexa^−/−^* and *Neu4^−/−^;Hexa^−/−^* mice. Values represent means ± S.D. of duplicate measurements performed on 3–11 mice of 4–12 month of age. * p<0.001 as compared with the *Hexa^−/−^* and *Neu4^−/−^;Hexa^−/−^* animals by the two-tailed nonparametric t-test. **Inset:** Representative TLC images of orcinol-stained gangliosides from brain of the *WT*, *Neu4^−/−^*, *Hexa^−/−^* and *Neu4^−/−^;Hexa^−/−^* mice at 12 months of age. Arrows indicate the positions of the ganglioside standards.

Bulk ganglioside composition of the brain tissues of 6 month-old *Neu4^−/−^;Hexa^−/−^* mice, as well as of their wild type littermates and those with single deficiencies of Neu4 or HexA were analyzed by thin-layer chromatography (TLC). The ganglioside composition in the brain of *Neu4^−/−^;Hexa^+/+^* mice did not differ from that of wild type animals with the exception of a previously reported decrease of brain G_M1_ ganglioside [10], whereas *Neu4^+/+^;Hexa^−/−^* animals showed remarkable storage of G_M2_ ganglioside, also consistent with previous reports [5], [6] (Figure 5). Although we have observed some variation in the content of different gangliosides among different animals with the same genotype, on average *Neu4^−/−^;Hexa^−/−^* mice showed a 2–3 fold higher ratio of G_M2_ to G_M1_ ganglioside as compared to *Neu4^+/+^;Hexa^−/−^* animals (Figure 5). This indicates that Neu4 is directly involved in desialylation of G_M2_ ganglioside because otherwise the Neu4 deficiency would proportionally decrease levels of both G_M1_ and G_M2_ gangliosides.

**Figure 6:**
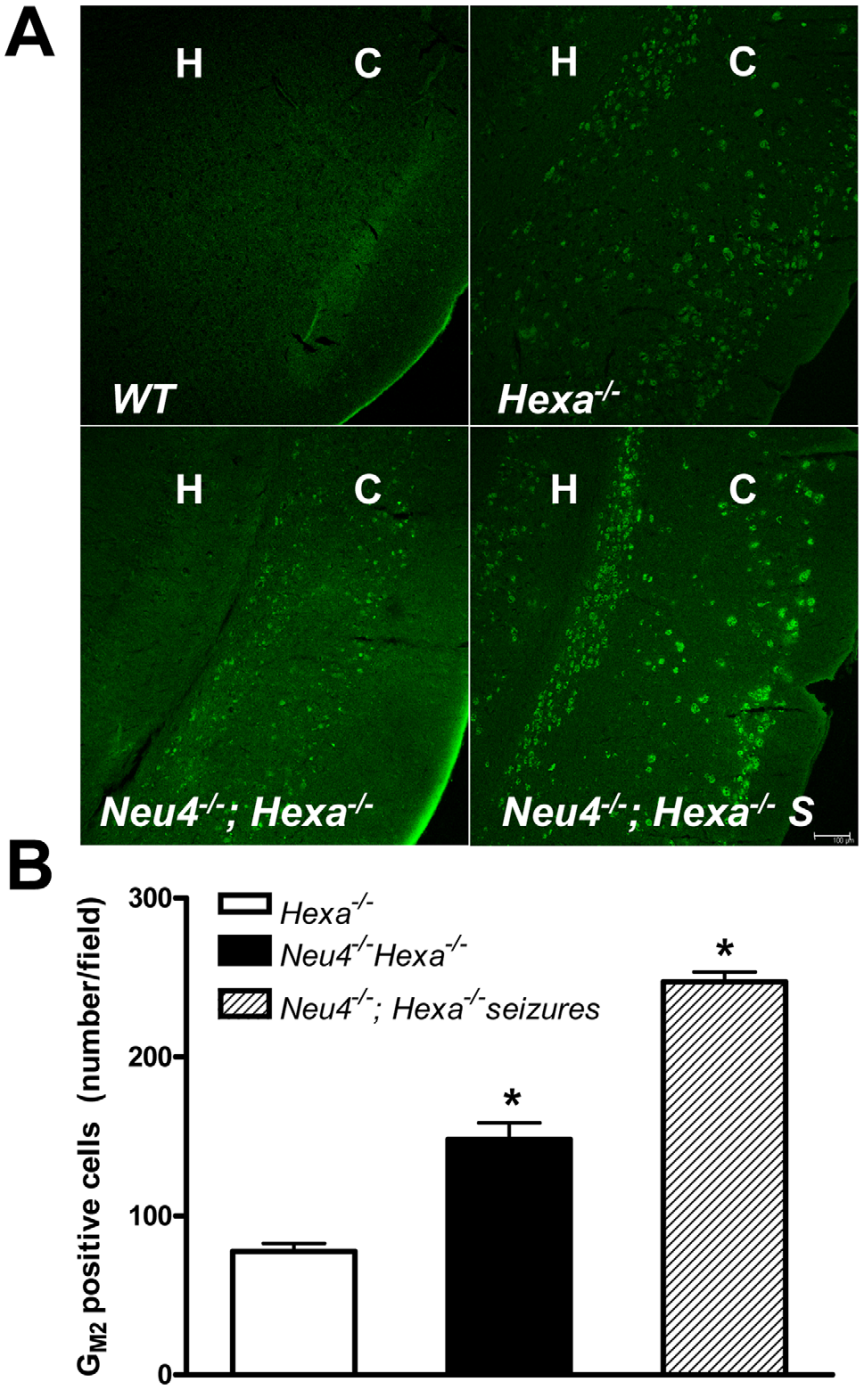
Increased accumulation of G_M2_ ganglioside in brain neurons of double knockout *Neu4^−/−^; Hexa^−/−^* mice. (**A**) Typical images of coronal sections from wild type (*WT*) mouse, mouse with HexA deficiency (*Hexa^−/−^*) and from double knockout mice without (*Neu4^−/−^;Hexa^−/−^*) and with episodes of seizures (*Neu4^−/−^;Hexa^−/−^ S*) stained with anti-G_M2_ antibodies. H indicates hippocampus, C – cortex. Scale bar: 100 µm. (**B**) Amount of neurons storing G_M2_-ganglioside in cortex from *Neu4^−/−^;Hexa^−/−^* mice with and without episodes of seizures and from a *Hexa^−/−^* mice. The cells were counted in 10 sections from similar cortical regions of each mouse. Three mice were studied for each group. * - significantly different from *Hexa^−/−^*.

**Figure 7:**
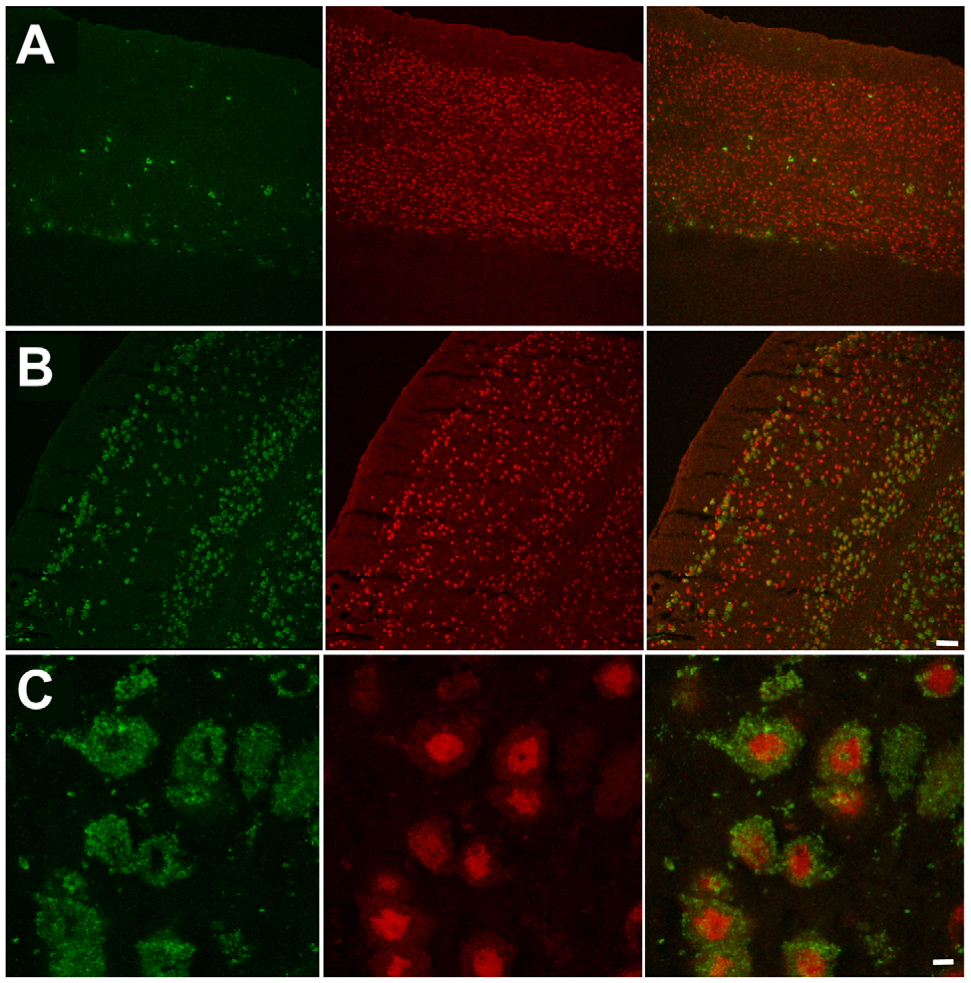
Numerous neurons in ventral cortex of double knockout, epileptic mouse store G_M2_.ganglioside. This panel shows G_M2_ (green, right panels) and NeuN (red, middle panels) immunostaining in dorsal (**A**) and ventral (**B**) cortex in a double knockout, epileptic mouse. G_M2_ positive neurons (yellow, right panels) are much more numerous in ventral cortex compared to dorsal cortex. Scale bar: 100 µm. (**C**) High magnification images showing G_M2_ (green, right panel) and NeuN (red, middle panel) immunostaining in layer 56 of ventral cortex in a double knockout, epileptic mouse. NeuN staining is stronger in the perinuclear region and weaker in the cytoplasm of neuronal somata. Scale bar: 5 µm. Panels represent typical images; 10 panels were studied.

Accumulation of G_M2_ ganglioside in brain cells was further studied by immunohistochemistry using the human-mouse chimeric monoclonal antibody, KM966 [16]. This antibody has been shown to be specific to G_M2_ ganglioside and is nonreactive toward glycolipid G_A2_ also present in *Neu4^+/+^;Hexa* ^−/−^ mice, toward globoside structurally similar to G_M2_ ganglioside, or toward G_M3_, G_D1a,b_ and G_D2a,b_ gangliosides [16], [17]. Analysis showed that G_M2_ ganglioside is undetectable in the brain of wild type or *Neu4^−/−^;Hexa^+/+^* mice, but present in the brains of *Neu4^+/+^;Hexa^−/−^* mice, as well as in double knockout *Neu4^−/−^*;*Hexa^−/−^* mice (Figure 6A). In both genotypes ganglioside storage was observed at low level in most areas of the brain, including the hippocampus, but was more prominent in deep layers of the ventral cortex (Figure 7A and 7B and Figures S11 and S12). Dual-labelling studies showed that G_M2_-accumulating cells are recognized by the NeuN antibody, indicating that they are neurons (Figure 7C). None of them, however, are recognized by an antibody against parvalbumin, a marker produced by about 50% of GABAergic interneurons, raising the possibility that G_M2_-accumulating cells are excitatory neurons (Figure S13).

In order to explore the relationship between G_M2_ ganglioside accumulation and seizure activity, we next compared the number of cells showing intense anti-G_M2_ antibody staining in the pyriform region of *Neu4^−/−^;Hexa^−/−^* and *Neu4^+/+^;Hexa^−/−^* mice (Figure 6B). *Neu4^−/−^*;*Hexa^−/−^* mice with seizures showed a significant increase of the number of G_M2_-storing cells (247±10) when compared with *Neu4^−/−^*;*Hexa^−/−^* mice without seizures (148±6) or with HexA deficient mouse (77±5). Accumulation of G_M2_ ganglioside in cortical neurons thus correlates with the occurrence of seizure in *Neu4^−/−^*;*Hexa^−/−^* mice.

## Discussion

Our study demonstrates that the Neu4 block exacerbates the neurological phenotype the *Hexa^−/−^* mouse, supporting the view that *Neu4* is a one of the modifier genes in the mouse model of Tay-Sachs disease. Neu4^/^Hexa double homozygotes showed a more severe disease than mice with either homozygous mutation alone. Double mutant mice were healthy at birth but developed seizures starting about 3 months of age, with EEG recordings showing typical epileptic events consisting of myoclonic jerks associated with spike wave discharges originating from the cortex and diffusing to the hippocampus, while in *Neu4* ^−/−^ and *Hexa* ^−/−^ mice seizures were not observed until at least one year of age. Seizures were observed in ∼40% of double-knockout mice which is consistent with a penetrence rate observed for seizures in several mouse models of Mendelian diseases [18], [19]. Interestingly, the seizures observed in double mutants were inducible by external stimuli such as handling and noise, which is reminiscent of the startle myoclonia that can be induced by noise in patients with Tay-Sachs disease. The exaggerated startle response found in Tay-Sachs disease, however, does not involve seizure activity but is likely explained by decreased central inhibition. The fact that noise can trigger myoclonic seizures in *Neu4^−/−^*;*Hexa^−/−^* mice suggests that decreased inhibition may likewise be a feature of these mice. In addition, *Neu4^−/−^*;*Hexa^−/−^* mice, but not the single mutants, showed subtle early signs of motor impairment, including tremor, weakness and spasticity, which can also be found in Tay-Sachs patients.

The more severe neurological impairment of *Neu4^−/−^*;*Hexa^−/−^* mice correlated with increased accumulation of G_M2_ ganglioside. This was confirmed by immunochemical analysis of their brain tissues using anti-G_M2_ antibodies which showed that double-knockout mice accumulate G_M2_ throughout the adult brain but more prominently in the ventral cortex and hippocampus. The number of G_M2_-positive cells was further increased in double-knockout mice with epileptic seizures. A similar correlation was observed between the severity of phenotype and the number of affected cortical and hippocampal neurons containing cytoplasmic vacuoles and storage bodies. Electron micrographs of the affected neurons showed large irregular lysosomes with characteristic membranous whorls closely resembling the histopathological findings in human Tay-Sachs patients. Interestingly the G_M2_-storing cells do not express parvalbumin, which labels approximately 50% of interneurons. Although we cannot exclude the possibility that these cells correspond to smaller populations of interneurons, it appears more likely that they are excitatory neurons. This latter interpretation suggests that the seizures observed in the double mutants are caused by the cortical lesions.

Our earlier *in situ* hybridization studies [10] indicated that Neu4 is expressed in scattered cells that are distributed throughout the adult brain, but with a greater density in the ventral cortex. The identity of these Neu4-expressing cells remains unknown. In the absence of Neu4 antibodies that are amenable to immunohistochemistry, we have used a combination of *in situ* hybridization and immunohistochemistry to show that Neu4-expressing cells are not microglial as we previously suggested [10] but now rule out or astrocytic cells (Figure S14). However, attempts at determining whether these cells are neurons failed because of the lack of neuron-specific antibodies that can function in the context of our dual-labeling approach. Interestingly, the number of G_M2_-accumulating cells in the ventral cortex of the double mutants appeared greater than the number of Neu4-expressing cells in the ventral cortex of wild type mice. This discrepancy could be explained by the possibility that Neu4 influences G_M2_ accumulation in a non-cell autonomous fashion. This hypothesis is consistent with our previous experiments showing that the enzyme secreted from transfected cells overexpressing Neu4 was taken up by neighbouring cells [11]. Alternatively, it is possible that Neu4 is expressed at lower levels in a greater number of cells but that expression at this level was not detectable by *in situ* hybridization.

If, however, Neu4 was the only sialidase contributing to the bypass of the HexA defect in *Hexa* ^−/−^ mice, then we would have anticipated a phenotype matching the severity observed in *Hexb* ^−/−^ mice. Since *Hexb* ^−/−^ mice lack both HexA and HexB, both arms of the G_M2_ catabolizing pathway are blocked. These mice show onset at 2–3 months of age with tremors, muscle weakness, stiffening of the hind limbs and an ataxic gait [5]–[6]. The disease rapidly progresses to spastic quadriparesis, tremor, myoclonus (startle and non-startle) and death by six weeks after onset. In our study the development of epileptic seizures and the accumulation of G_M2_ storage in adult *Neu4* ^−/−^;*Hexa* ^−/−^ mice demonstrates the predicted increased severity of disease indicative of the importance of Neu4, but it is clear from the much milder than expected phenotype that other sialidases also contribute to the bypass. Two other sialidases, Neu1 and Neu3, are also abundant in the mouse brain. Neu1 is a component of a lysosomal multienzyme complex consisting of Neu1, a protective protein/cathepsin A (PPCA) and β-galactosidase (reviewed in [20]). Neu1 shows *in vitro* G_M3_ and G_D1a_ ganglioside hydrolyzing activity, but weak activity toward G_M2_ and is most active toward sialyloligosaccharides and sialylglycoproteins [10], [21]. Impaired metabolism of G_M3_ ganglioside was observed in cultured skin fibroblasts from patients with primary (sialidosis) or secondary (galactosialidosis) sialidase deficiency [22] as well as a storage of G_M3_ and G_D3_ gangliosides in visceral tissues but not in brain of sialidosis patients [23]. Neu3 sialidase abundantly expressed in mouse cerebellum [24] is present at the plasma membrane and has also been described as facing inward on the membranes of endosomes and lysosomes [25]. It shows high activity toward majority of gangliosides including G_M2_ similar to that of Neu4 [10]–[11], [14]. Further studies involving generation of genetically targeted mice deficient in other neuraminidases and their breeding with *Hexa^−/−^* mouse are therefore necessary to determine which of these enzymes contributes, along with Neu4, to the bypass in *Hexa* ^−/−^ mice.

Our mouse model with a double HexA/Neu4 deficiency progresses toward the neuropathological abnormalities of *Hexb^−/−^* mice or of human Tay-Sachs patients. While other sialidases also may contribute to the bypass pathway, it is important to note that transfection of Neu4 in cultured fibroblasts from patients with sialidosis or galactosialidosis and of neuroglia cells from a patient with Tay-Sachs disease resulted in increased sialidase activity and normalization of lysosomal morphology [10], [11]. Also, correction was observed in cells not receiving the targeting vector, suggesting that secretion and reuptake of Neu4 might also contribute to disease amelioration. Because Neu4 targets to the lysosome, but does not require formation of a multienzyme complex and appears to participate in secretion and reuptake by nearby cells, it might act as an optimal pharmacologic modifier, perhaps through pharmacologic induction, for the treatment of human Tay-Sachs disease.

## Materials and Methods

### Animals

Generation of mice with targeted disruption of *Neu4* and *Hexa* genes has been previously described [6], [10]. Both strains were back-crossed for at least 5 generations to C57BL/6NCrl strain distributed by Charles River Quebec. Homozygous animals from each genotype were bred to each other and the litter genotyped by PCR as previously described. Expression of the *Neu4* and *HexA* genes in mouse tissues was also analyzed by RT-PCR as described below. The Neu4-deficient mice were compared with the appropriate littermate controls. All mice were bred and maintained in the Canadian Council on Animal Care (CCAC)-accredited animal facilities of the Ste-Justine Hospital Research Center according to the CCAC guidelines. Mice were housed in an enriched environment with continuous access to food and water, under constant temperature and humidity, on a 12 h light:dark cycle. Approval for the animal care and the use in the experiments was granted by the Animal Care and Use Committee of the Ste-Justine Hospital Research Center.

### Genotyping of mice

The genotypes of mice were determined using genomic DNA extracted from the clipped tail tip. The PCR was performed in a total volume of 50 µl containing 100 pmol of each primer, 0.2 mM dNTPs, 1.5 U taq polymerase (Invitrogen) and 100 ng genomic DNA in 20 mM Tris (pH 7.4), 50 mM KCl, and 1.5 mM MgCl_2_. Multiplex primers for detection of *Hexa* alleles were 5′-GGCCAGATACAATCATACAG (Hexa-F), 5′-CTGTCCACATACTCTC CCCACAT (Hexa-R), 5′-CACCAAAGAAGGGAGCCGGT (PGK) and for the detection of *Neu4* alleles were 5′-CTCTTCTTCATTGCCGTGCT (Neu4F), 5′-GCCGAATATCATGGTGGAAA (Neu4R), 5′-GACAAGGAGAGCCTC TGGTG (Neo). Samples were denatured for the first cycle at 95°C for 5 min, followed by 35 cycles at 94°C for 20 s, 57°C for 20 s and 72°C for 1 min, with a final extension reaction at 72°C for 10 min. Similar PCR conditions were used for *Hexa* and *Neu4* allele genotyping.

### Quantitative RT-PCR

Total RNA was isolated from cultured cells or mouse tissues using the Trizol Reagent (Invitrogen) according to the manufacturer's protocol and reverse-transcribed to cDNA by using random primers and SuperScript III reverse transcriptase (Invitrogen). Quantification of mouse *Neu4* mRNA in cultured cells and mouse tissues was performed using a LightCycler system (Roche, Germany) and the following set of primers: 5′-TGC AGT ACT GGA GGA GCA CA-3′ and 5′-AGG TGT AAG CAG GAA CAA GCA-3′. β-Actin mRNA was used as a reference control.

### Neurological examination of mice

The motor performance of mice was evaluated using a simplified neurological examination as previously described [26], [27]. The evaluation was composed of the following tests: activity (the normal mouse is placed in a clean cage with clean bedding animals and must explore 3 sides of the cage within 1 min), locomotion (visual appreciation of locomotion, evaluation of motor coordination i.e. normal linear progression, stance and tremors), visual positioning (when placed approximately 10 cm above a solid surface and lowered slowly toward the surface, the capacity to symmetrically extend legs with a normal locomotor pattern is evaluated), climbing (ability to climb up a gridded surface with 1 cm squares) and force (ability to hang on the side of the cage with 2 front legs when the mouse is placed in contact with cage).

### Monitoring for seizures

Electroencephalograms (EEGs) were performed in mice. A stainless steel bipolar electrode (Plastics one, Roanoke, VA) was chronically implanted, through a burr hole, into the right dorsal hippocampus and overlying cortex. After a week recovery period, EEGs were recorded for 2 hours per day for 4 to 5 days with the Stellate Harmonie video-EEG system (Stellate, Montreal, Quebec, Canada). Proper electrode placement within the target structures was confirmed in all mice.

### Rotating rod

The rotating rod motor coordination test was performed using an accelerating Rota-rod treadmill for mice (3 cm diameter) to assess for motor deficits. Animals were briefly trained before test at 4 rpm on a 5 line rota-rod unit. The animals were then tested using an accelerated mode from 4 to 40 rpm over 5 minutes. Three trials were performed, each separated by a 20 minute rest period. The length of time that each animal was able to stay on the rod was recorded.

### Open field test

The open field test was performed to assess behavioural disturbances associated with cortical dysfunction in mice [15]. The apparatus consisted of an arena of 40×40 cm in surface area and was surrounded from all sides by a 50 cm transparent wall. A digital camera was mounted directly above the apparatus. The images were transmitted to a PC running Any-maze tracking system. Mice were gently placed in the center of the arena and allowed to explore undisturbed for 15 minutes on the first day (exploratory phase) and 3 days after habituation (habituation phase). The observed parameters were indexed by traveled distance recorded during 15 min and numbers of grooming and rearing.

### Morris Water Maze test

The mice were subjected to the Morris Water Maze test for spatial learning [28] at the age of 4 months. Mice first received a 3-day habituation period requiring them to swim (60 s) to a visible platform located in a circular tank (1.4 m in diameter) filled with water made opaque with inert paint (25±1°C). Then wall visual cues and platform location were switched, the platform was submerged (1 cm), and 5 days of hidden-platform trials ensued. Two hours following hidden-platform testing, all mice were given a probe trial (60 s) in which percentage time spent and distance traveled in the target quadrant (no longer containing a platform) were recorded, along with swim speed. In the hidden platform testing, mice were given 5 trials of 90 s to find the platform (maximum inter-trial interval of 45 min), being guided to and allowed to stay on the platform for 5 s on the first day if they exceeded the allotted time. Visual acuity and motivation were tested during the habituation period. Escape latencies were acquired with the 2020 Plus tracking system and Water 2020 software (Ganz FC62D video camera; HVS Image, UK). Animals were allowed to dry under a heating lamp after each trial to avoid hypothermia, and all experiments were started at the same time every day. All experiments were performed by the same investigator (VS).

### *In situ* hybridization

Newborn brains were fixed in the Carnoy's fluid, embedded in paraffin and sectioned at 6 µM. Seven week-old mice were anaesthetised and perfused via cardiac puncture with saline and then 4% paraformaldehyde. Whole brains were rapidly removed, post-fixed overnight and then processed to generate four sets of 20 µM adjacent coronal sections with a cryostat. *In situ* hybridization was performed using radioactive probes as previously described [10] on one out of two sections of newborn brain sets and selected adjacent sections from adult brains at an interval of 150 µM. The *Neu1* probe contained the entire 1.2 kb of mouse cDNA. The *Neu4* probes corresponding to a 1.1-kb sequence fragments were generated by RT-PCR, using mouse newborn total brain RNA extracts and the following set of primers: 5′-AAG CTT GAC TGG GCC ACC TTT GCT-3′ and 5′-CTG CAG GCC AGC AAT GCC CCT GA-3′. The RNA probes were transcribed from linearized plasmids using either T7 or T3 RNA polymerase in the presence of [^35^S]-UTP. Both sense and antisense transcripts were used. After hybridization, slides were dipped in the emulsion and exposed for a period of 2 weeks.

### Immunohistochemistry

To prepare fixed tissues for immunohistochemical analysis, transcardiac perfusion was initiated with phosphate–buffered saline (pH 7.4) followed by 4% paraformaldehyde in phosphate-saline buffer. Brain tissues were removed and placed in the same fixative overnight at 4°C, and then treated sequentially in 10%, 20% and 30% sucrose in 0.1 M sodium phosphate buffer (pH 7.2) overnight at 4**°**C. The tissue blocks were embedded in OCT compound before freezing at −80**°**C. Ten-twelve µm thick sections were cut in cryostat and collected onto poly-lysine coated slides. The slides were treated with 0.1% Triton X-100, blocked in a humidified chamber with 5% goat serum, and KM966 antibody, 1∶600 in 1XPBS, to detect G_M2_ ganglioside. The slides were further incubated with DyLight 488-conjugated Affinipure Goat anti-human IgG (Jackson immunoresearch laboratories) and mounted with Cytoseal mounting medium. Slides were studied on a Zeiss LSM510 inverted confocal microscope (Zeiss). Images were processed and quantified using the LSM image browser software (Zeiss) and Photoshop (Adobe).

### Enzyme assays

Sialidase, β-glucosidase and β-hexosaminidase A activities in cellular homogenates and in subcellular fractions from brain tissues of *Neu4^−/−^* and wild type littermates mice were assayed using the corresponding fluorogenic 4-methylumbelliferyl glycoside substrates as previously described [10]. Protein concentration was determined using a Bio-Rad Bradford kit.

### Light microscopy of mouse tissues

Knockout mice and their wild type littermates, 6 months of age, were anesthetized with sodium pentobarbital and the brains fixed by perfusion with 4% paraformaldehyde in phosphate-buffered saline. After perfusion the brains were carefully removed, sliced by a coronal section through the midregion, and immersed in the same fixative for an additional 24 hrs. The tissues were dehydrated in graded ethanol and embedded in paraffin. Paraffin sections (5 µm thick) were deparaffinized in toluene, rehydrated with ethanol (90%–50%) and stained with hematoxylin and eosin. The sections were viewed and photographed in a Leica light microscope.

### Quantitative analysis of affected neurons in mouse brain

The cerebral cortex of mice from each group was subdivided into superficial and deep regions. Adjacent superficial and deep areas were photographed for manual counting of vacuolated neurons by a non-biased observer blinded for the genotype of mice. Nine epon sections for cortex and 9 sections for hippocampus were analyzed for each mouse and 3 mice were studied for each genotype.

### Electron microscopy of mouse tissues

For electron microscopy evaluation knockout mice and their wild type littermates, 6 months of age, were anesthetized with sodium pentobarbital and the brains were perfused with 2.5% glutaraldehyde in 5 mM phosphate, pH 7.5. The brains were then carefully removed and a coronal section (2 mm thick) containing the cortex and hippocampus was obtained using a razor blade. The cortex and hippocampus were further trimmed into small cubes and immersed in the same fixative for an additional 24 hs. The tissues were dehydrated in graded ethanol and propylene oxide and embedded in epon. Semi-thin sections were cut and mounted on glass slides, stained with toluidine blue and viewed by light microscopy. The regions of interest for electron microscopy were selected and ultrathin sections were cut and mounted on 200 mesh copper grids. Staining of the grids was done with uranyl acetate for 5 min, followed by lead citrate for 2 min. The grids were viewed on a Tecnai FEI electron microscope.

### Measurement of gangliosides in mouse tissues

Lipids were extracted by adding 1 ml of a chloroform/methanol mixture (1∶1, v/v) to 250 mg aliquots of frozen brain tissue lysates followed by homogenization using a Polytron PT3000 (Brinkman). Phase separation was induced by adding 0.65 ml of phosphate-buffered saline (PBS). After centrifugation at 500 g for 15 min, the upper phase containing gangliosides was isolated. The lower phase was washed first with PBS and then with water. The upper phases were combined and passed through a Supelclean LC-18 column (Supelco). Gangliosides were eluted first using methanol and then the chloroform/methanol mixture. After evaporation under a stream of nitrogen, the residue was resuspended in 0.1 ml of the chloroform/methanol mixture and applied to a silica gel thin-layer chromatography (TLC) plate that was developed using chloroform/methanol/0.22% CaCl_2_ (55∶45∶10, by vol.). After staining with orcinol or resorcinol, gangliosides were identified by comparing their Rf to those of authentic porcine brain ganglioside standards (Avanti Polar Lipids).

### Statistical analysis

Statistical analysis was performed using two-tailed t-test and ANOVA test.

## Supporting Information

Figure S1 **Genotyping of** ***Hexa*** **-knockout and** ***Neu4*** **-knockout mice by PCR analysis of tail genomic DNA.** (A) *Hexa* allele-specific PCR amplifying a 420 bp fragment in wild type (+/+) mice, 420 and 210 bp fragments in heterozygous (+/−) mutants and 210 bp fragment in homozygous (−/−) HexA-deficient animals. (B) *Neu4* allele-specific PCR amplifying a 465 bp fragment in wild-type (+/+) mice, 465 and 635 bp fragments in heterozygous (+/−) mutants and 635 bp fragment in homozygous (−/−) Neu4-deficient animals.
(0.02 MB PDF)

Figure S2 **Fold induction of four neuraminidase genes (** ***Neu1-Neu4*** **) in adult brains of wild type,** ***Neu4^−/−^*** **,** ***Hexa^−/−^*** **and** ***Neu4^−/−^;Hexa^−/−^*** **mice.** No presence of *Neu4* transcripts was detected in the brains of *Neu4^−/−^* and *Neu4^−/−^;HexA^−/−^* mice. Data show the means ± SD of 2 independent experiments; brains of 3 mice were studied for each genotype. Data were normalised to the level of β–actin and expressed as fold increase as compared to the expression levels in wild type mouse.
(0.01 MB PDF)

Figure S3 **Confirmation of β-hexosaminidase A deficiency in brain tissues from single knockout** ***Hexa^−/−^*** **and double-knockout** ***Neu4^−/−^;Hexa^−/−^*** **mice.** HexA activity in total brain was measured against 4-metylumbelliferyl-N-acetylglucosamine-6-sulfate (MUGS) substrate at pH 4.2 as described in Phaneuf et al. (1996; Hum Mol Genet 5: 11–14). Data represent the mean ± SD of three independent experiments performed on brains from 3 different mice for each genotype. * - statistically different (p<0.05) from the WT group.
(0.01 MB PDF)

Figure S4 **Paralysis on the right hind limb in the 4-months old** ***Neu4^−/−^;Hexa^−/−^*** **mouse.**
(0.03 MB PDF)

Figure S5 **At the age of 4 months double-knockout** ***Neu4^−/−^*** **;** ***Hexa^−/−^*** **mice show similar performance on the rotarod test as single knockout** ***Hexa^−/−^*** **mice.** Graph shows the means ± SD. Six mice were studied for each genotype.
(0.01 MB PDF)

Figure S6 **At the age of 4 months both** ***Hexa^−/−^*** **and** ***Neu4^−/−^*** **;** ***Hexa^−/−^*** **mice showed a similar traveled distance and adaptation rate in the open field test when compared at day 5.** Graph shows the means ± SD. Six mice were studied for each genotype.
(0.01 MB PDF)

Figure S7 **Neither** ***Hexa^−/−^*** **nor** ***Neu4^−/−^*** **;** ***Hexa^−/−^*** **mice showed impaired performance in the spatial memory-based Morris Water Maze test at 4 months.** All mice showed a similar average latency on day 1 of a hidden platform test and a similar learning curve. Only the data from trials on days 4–8 that consisted of the hidden platform testing are shown. Graph shows the means ± SD. Six mice were studied for each genotype.
(0.01 MB PDF)

Figure S8 **During the removed-platform probe trial on day 8, wild type,** ***Hexa^−/−^*** **and** ***Neu4^−/−^*** **;** ***Hexa^−/−^*** **mice displayed close time and traveling distance in the target quadrant.** Numbers of passes through the removed platform were also similar. Swim speed was comparable among all groups. Graph shows the means ± SD. Six mice were studied for each genotype.
(0.01 MB PDF)

Figure S9 **Light microscopy of neurons in wild type mice (WT), singe** ***Neu4^−/−^*** **or** ***Hexa^−/−^*** **knockouts and in double knockouts without (** ***Neu4^−/−^;Hexa^−/−^*** **) and with seizures (** ***Neu4^−/−^;Hexa^−/−^ S*** **).** Affected neurons containing vacuolated cytoplasm are shown by arrows. **H** indicates hippocampus, **C** – cortex. The panels show representative images of at least 20 panels studied for 3 mice in each group.
(0.15 MB PDF)

Figure S10 **Electron micrographs of hippocampal neurons from wild type (A), single** ***Neu4^−/−^*** **(B) and** ***Hexa^−/−^*** **(C) knockouts, and double (** ***Neu4^−/−^;Hexa^−/−^*** **) knockouts without (D) and with seizures (E).** Affected neurons containing vacuolated cytoplasm are only present in B, C and D. Bars range between 1 and 2 µm. The panels show representative images of at least 20 panels studied for 3 mice in each group.
(0.34 MB PDF)

Figure S11 **G_M2_ ganglioside is stored by sparse neurons in CA1 region of the hippocampus of** ***Neu4^−/−^;Hexa^−/−^*** **mouse with seizures.** **(A)** G_M2_ ganglioside (green, right panels) and NeuN (red, middle panels) immunostaining in CA1 region of the hippocampus in a *Neu4^−/−^;Hexa^−/−^* mouse with seizures. NeuN staining is stronger in the perinuclear region of neuronal somata. **(B)** High magnification images showing that G_M2_ ganglioside is stored by neurons in the pyramidal layer (arrows). Scale bars: A, 50 µm; B, 20 µm. The panels show representative images of at least 15 panels studied for 3 mice in each group.
(0.52 MB PDF)

Figure S12 **G_M2_ ganglioside is stored by sparse neurons in CA3 and dentate gyrus regions of the hippocampus of double KO, epileptic mouse.** Panels show G_M2_ ganglioside (green, right panel) and NeuN (red, middle panels) immunostaining in CA3 (**A**) and dentate gyrus (**B**) regions of the hippocampus in a double *Neu4−/−;Hexa−/−* mouse with seizures. G_M2_ colocalizes with NeuN (arrows). Scale bar: 100 µm. Panels show representative images of at least 15 panels studied for 3 mice in each group.
(0.54 MB PDF)

Figure S13 **G_M2_ ganglioside is not stored by parvalbumin-positive GABAergic interneurons in cortex of** ***Neu4^−/−^;Hexa^−/−^*** **mouse with seizures.** Panels show G_M2_ ganglioside (green, right panel) and NeuN (red, middle panels) immunostaining in dorsal (**A**) and ventral (**B**) cortex in a *Neu4^−/−^;Hexa^−/−^* mouse with seizures. Note that Parvalbumin-positive neurons do not store G_M2_ (arrows). Parvalbumin is expressed by about 50% of cortical GABAergic interneurons. Scale bar: 100 µm.
(0.39 MB PDF)

Figure S14 **Neu4 is not expressed by astrocytes or microglia cells.** *In situ* hybridization with a Neu4 probe labels few sparse cells in CA1 region of the hippocampus (arrows, left and right panels). These cells are negative for immunostaining for the astrocyte marker GFAP (**A**, green, middle panel) and microglial marker Eba1a (**B**, red, middle panel) Scale bar: 20 µm. The panels show representative images of at least 10 panels studied.
(0.36 MB PDF)

Video S1 (4.73 MB WMV)
