# Supplementary figures and images for: Mice Doubly-Deficient in Lysosomal Hexosaminidase A and Neuraminidase 4 Show Epileptic Crises and Rapid Neuronal Loss
Source: PLoS Genet. 2010 Sep 16;6(9):e1001118. doi: 10.1371/journal.pgen.1001118 (PMC2940724; doi:10.1371/journal.pgen.1001118)

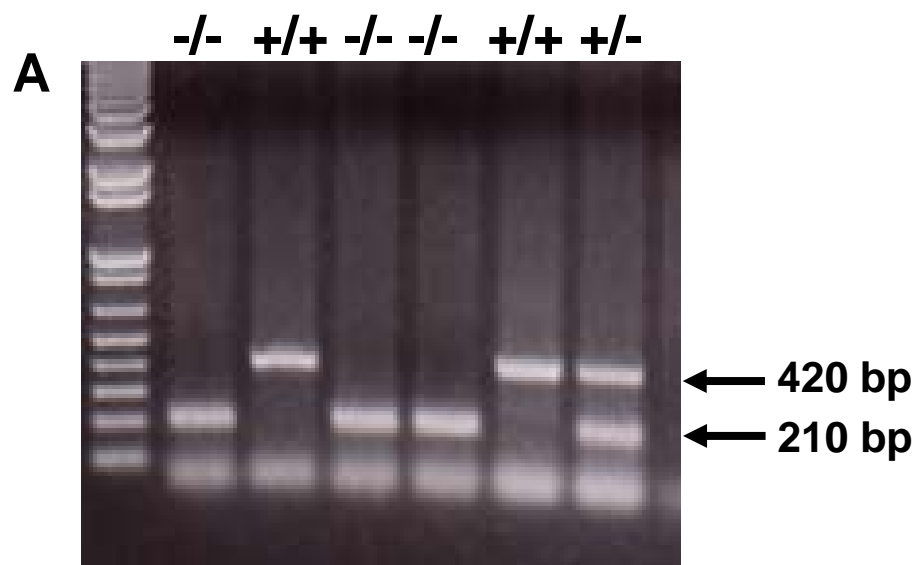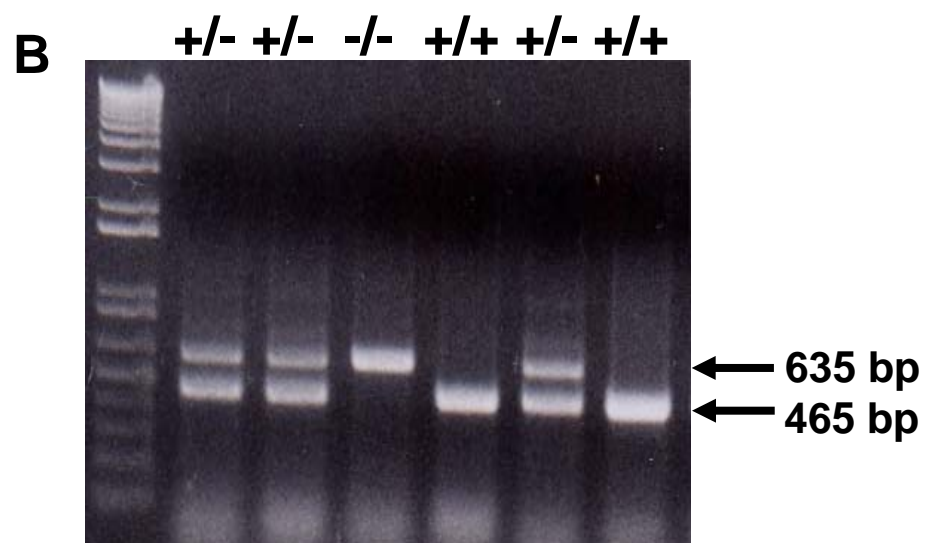

Supplement: Figure S1 — Genotyping of Hexa -knockout and Neu4 -knockout mice by PCR analysis of tail genomic DNA. (A) Hexa allele-specific PCR amplifying a 420 bp fragment in wild type (+/+) mice, 420 and 210 bp fragments in heterozygous (+/−) mutants and 210 bp fragment in homozygous (−/−) HexA-deficient animals. (B) Neu4 allele-specific PCR amplifying a 465 bp fragment in wild-type (+/+) mice, 465 and 635 bp fragments in heterozygous (+/−) mutants and 635 bp fragment in homozygous (−/−) Neu4-deficient animals. (0.02 MB PDF) [file pgen.1001118.s001.pdf]

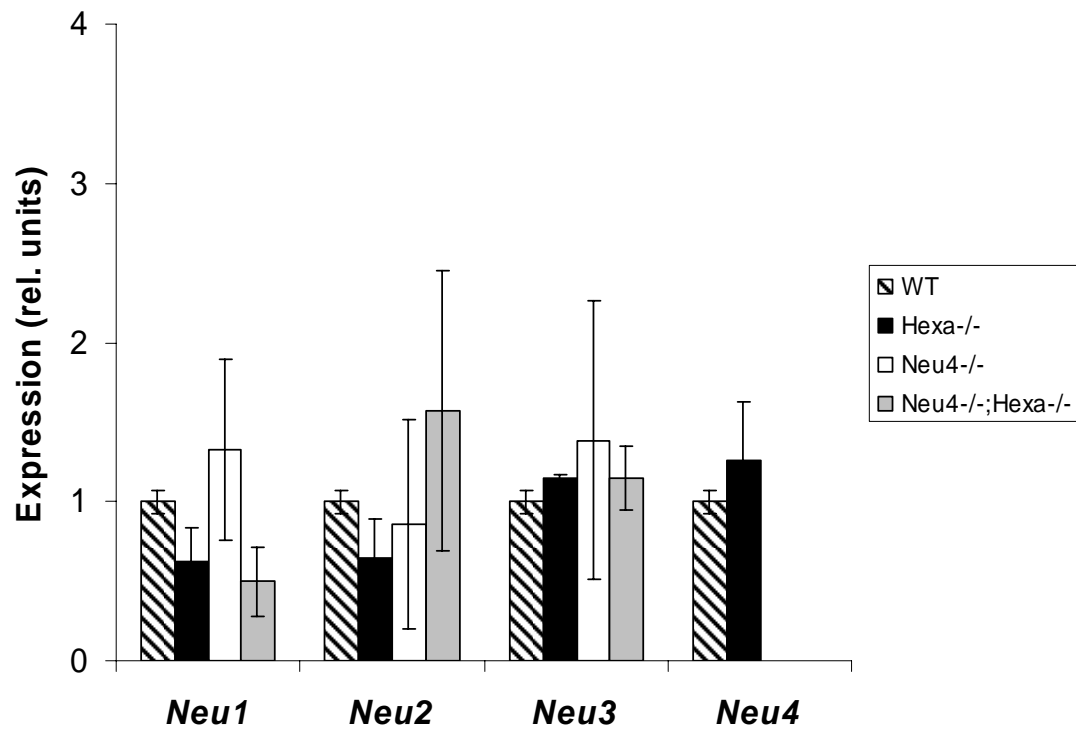

Supplement: Figure S2 — Fold induction of four neuraminidase genes ( Neu1-Neu4 ) in adult brains of wild type, Neu4−/− , Hexa−/− and Neu4−/−;Hexa−/− mice. No presence of Neu4 transcripts was detected in the brains of Neu4−/− and Neu4−/−;HexA−/− mice. Data show the means ± SD of 2 independent experiments; brains of 3 mice were studied for each genotype. Data were normalised to the level of β–actin and expressed as fold increase as compared to the expression levels in wild type mouse. (0.01 MB PDF) [file pgen.1001118.s002.pdf]

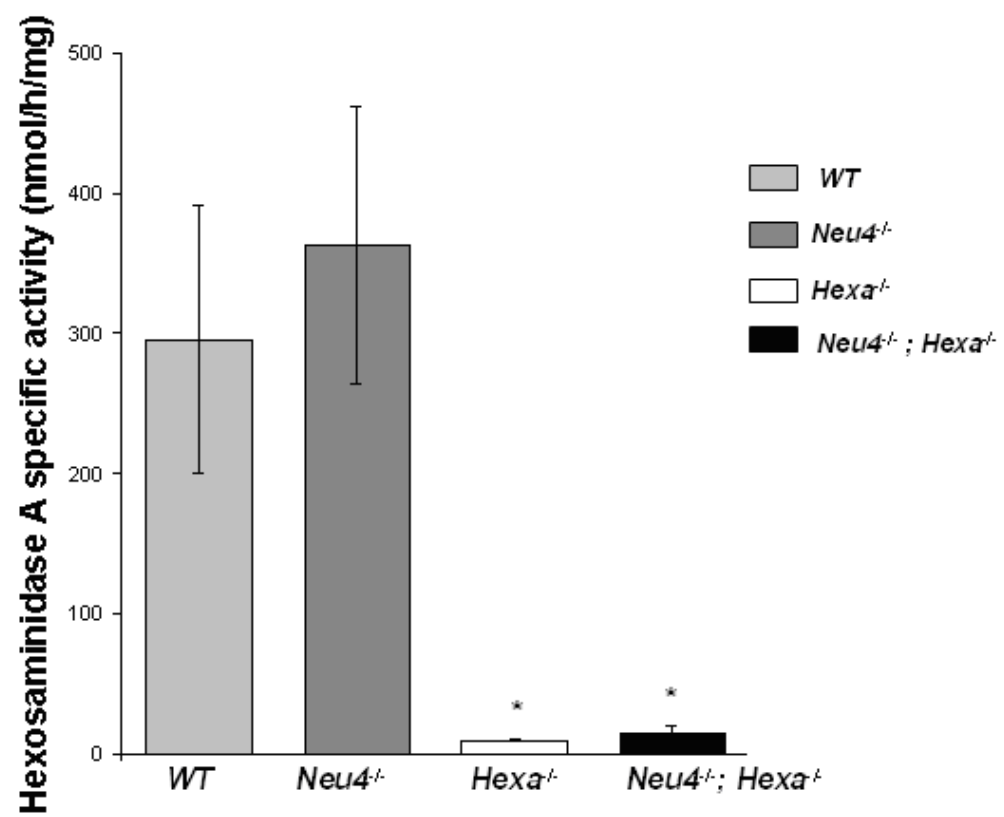

Supplement: Figure S3 — Confirmation of β-hexosaminidase A deficiency in brain tissues from single knockout Hexa−/− and double-knockout Neu4−/−;Hexa−/− mice. HexA activity in total brain was measured against 4-metylumbelliferyl-N-acetylglucosamine-6-sulfate (MUGS) substrate at pH 4.2 as described in Phaneuf et al. (1996; Hum Mol Genet 5: 11–14). Data represent the mean ± SD of three independent experiments performed on brains from 3 different mice for each genotype. * - statistically different (p<0.05) from the WT group. (0.01 MB PDF) [file pgen.1001118.s003.pdf]

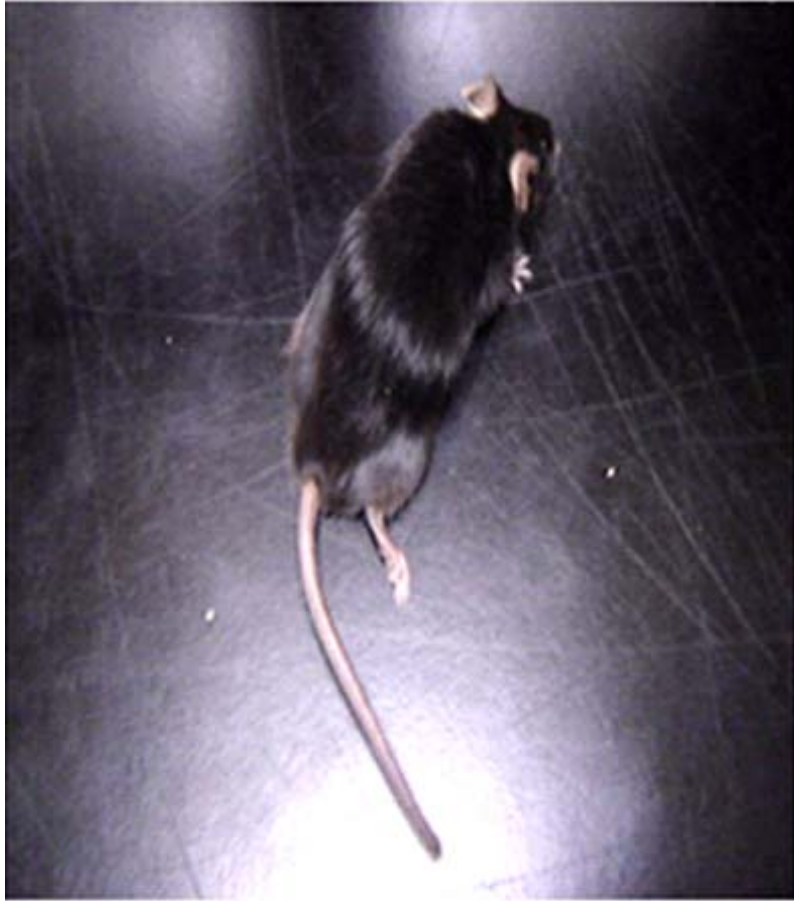

Supplement: Figure S4 — Paralysis on the right hind limb in the 4-months old Neu4−/−;Hexa−/− mouse. (0.03 MB PDF) [file pgen.1001118.s004.pdf]

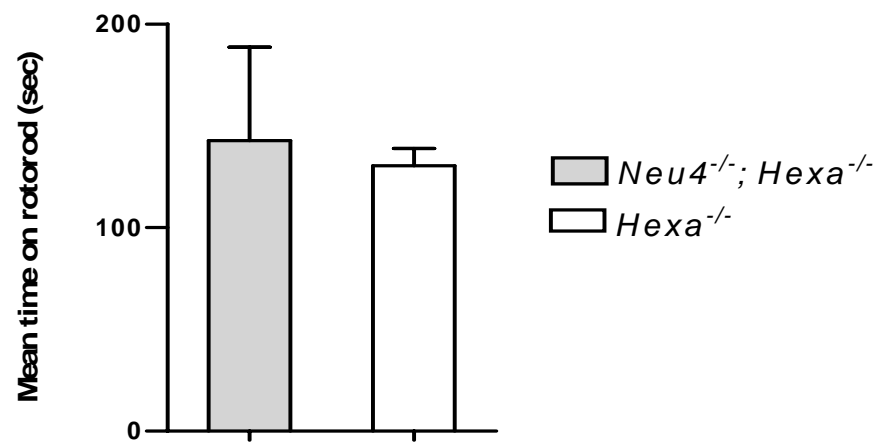

Supplement: Figure S5 — At the age of 4 months double-knockout Neu4−/− ; Hexa−/− mice show similar performance on the rotarod test as single knockout Hexa−/− mice. Graph shows the means ± SD. Six mice were studied for each genotype. (0.01 MB PDF) [file pgen.1001118.s005.pdf]

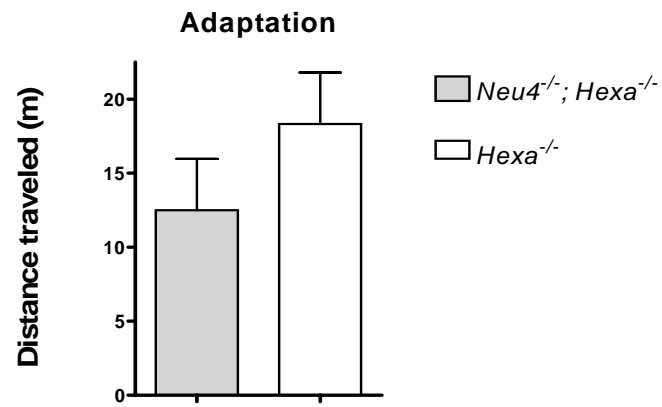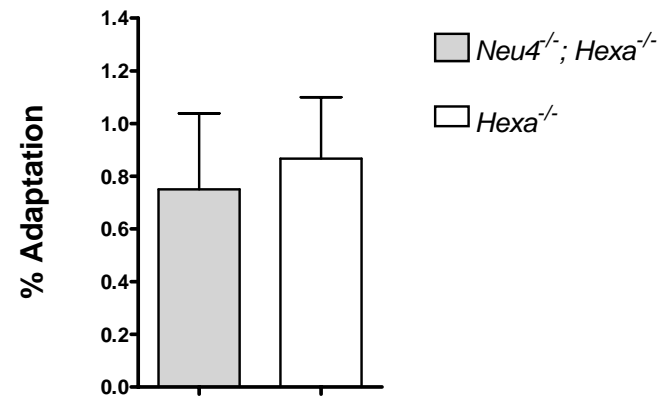

Supplement: Figure S6 — At the age of 4 months both Hexa−/− and Neu4−/− ; Hexa−/− mice showed a similar traveled distance and adaptation rate in the open field test when compared at day 5. Graph shows the means ± SD. Six mice were studied for each genotype. (0.01 MB PDF) [file pgen.1001118.s006.pdf]

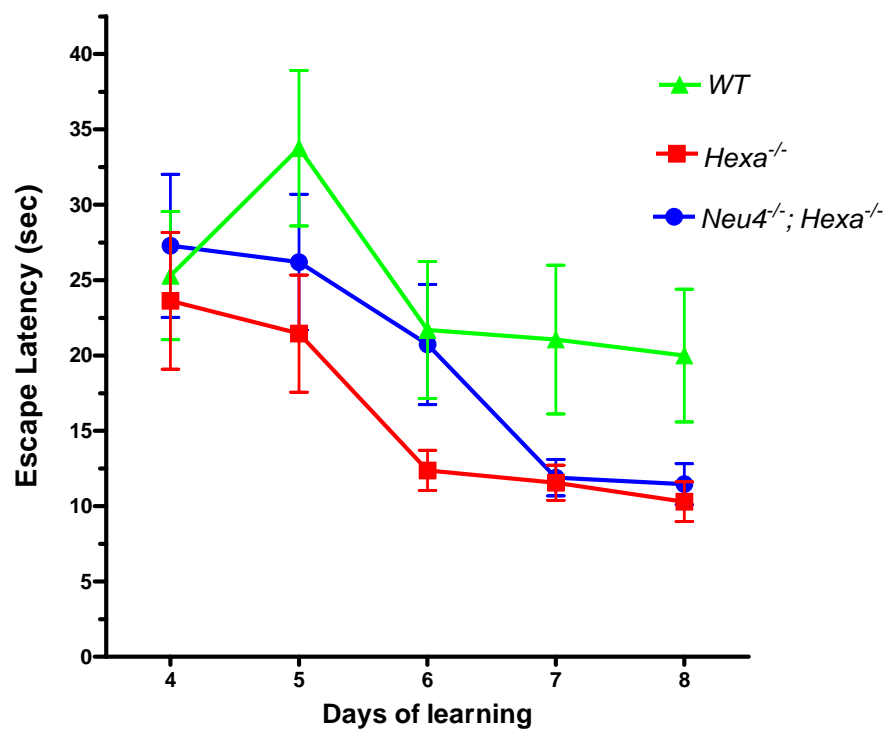

Supplement: Figure S7 — Neither Hexa−/− nor Neu4−/− ; Hexa−/− mice showed impaired performance in the spatial memory-based Morris Water Maze test at 4 months. All mice showed a similar average latency on day 1 of a hidden platform test and a similar learning curve. Only the data from trials on days 4–8 that consisted of the hidden platform testing are shown. Graph shows the means ± SD. Six mice were studied for each genotype. (0.01 MB PDF) [file pgen.1001118.s007.pdf]

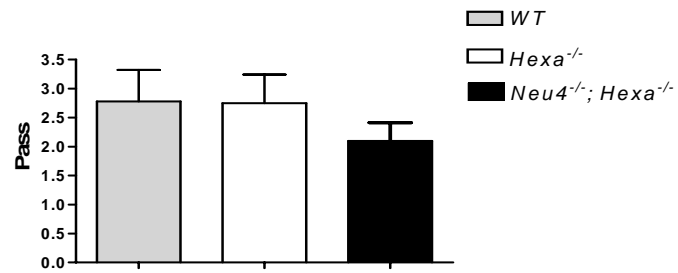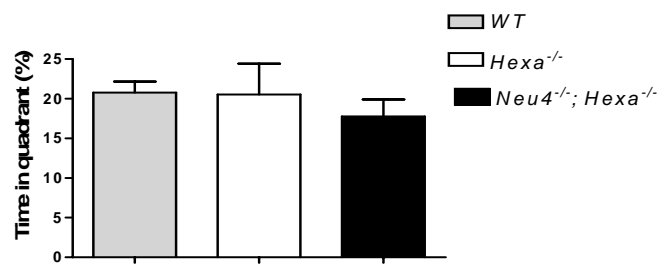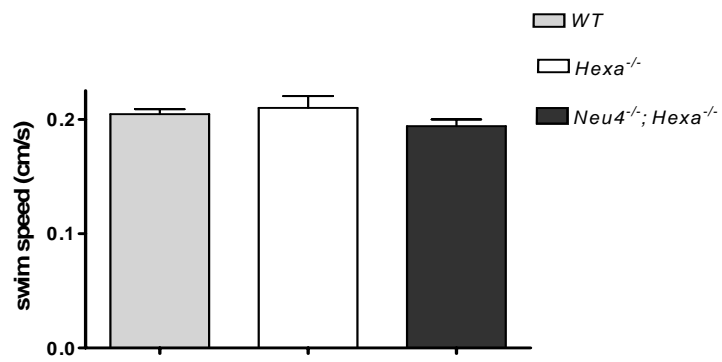

Supplement: Figure S8 — During the removed-platform probe trial on day 8, wild type, Hexa−/− and Neu4−/− ; Hexa−/− mice displayed close time and traveling distance in the target quadrant. Numbers of passes through the removed platform were also similar. Swim speed was comparable among all groups. Graph shows the means ± SD. Six mice were studied for each genotype. (0.01 MB PDF) [file pgen.1001118.s008.pdf]

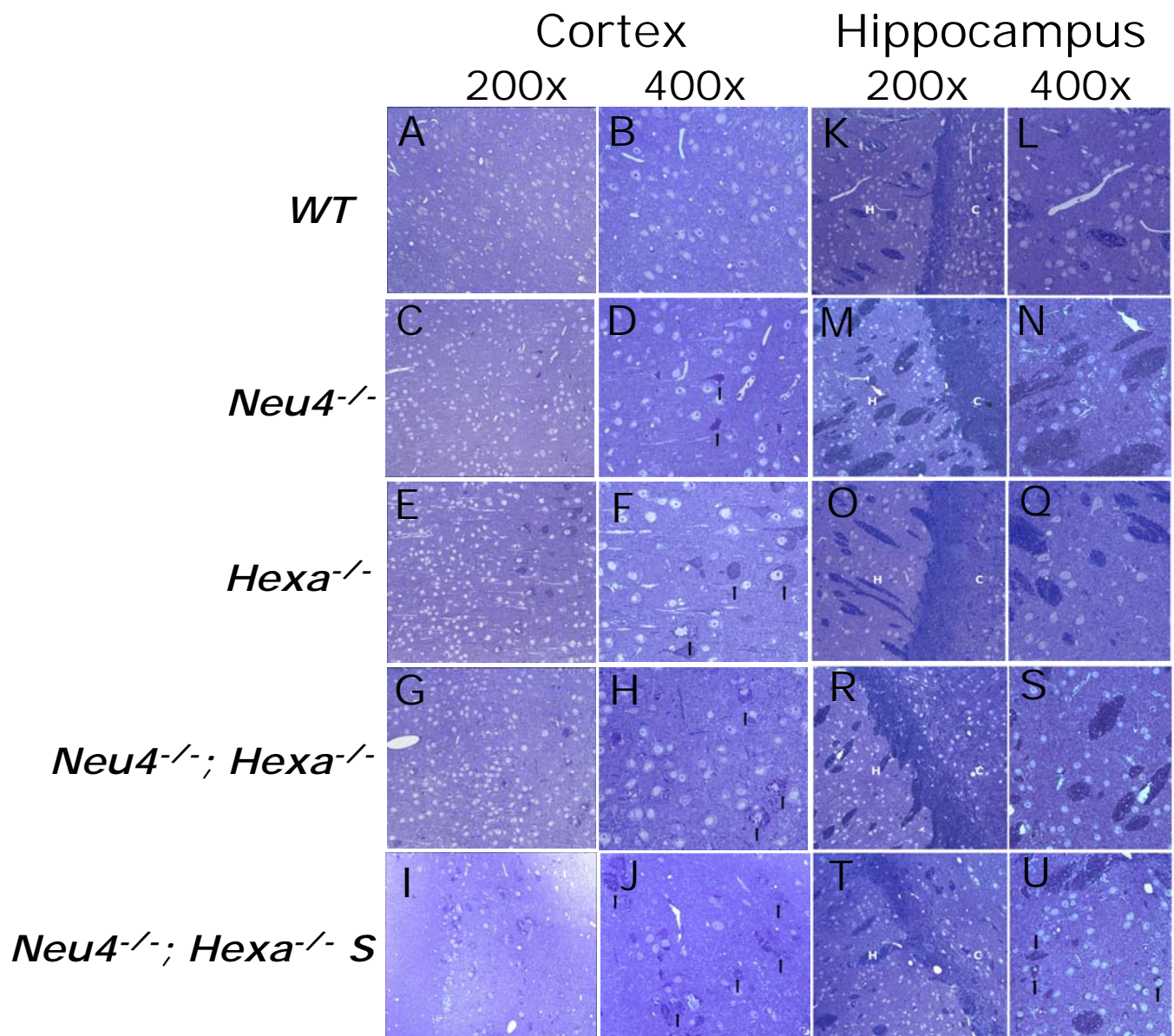

Supplement: Figure S9 — Light microscopy of neurons in wild type mice (WT), singe Neu4−/− or Hexa−/− knockouts and in double knockouts without ( Neu4−/−;Hexa−/− ) and with seizures ( Neu4−/−;Hexa−/− S ). Affected neurons containing vacuolated cytoplasm are shown by arrows. H indicates hippocampus, C – cortex. The panels show representative images of at least 20 panels studied for 3 mice in each group. (0.15 MB PDF) [file pgen.1001118.s009.pdf]

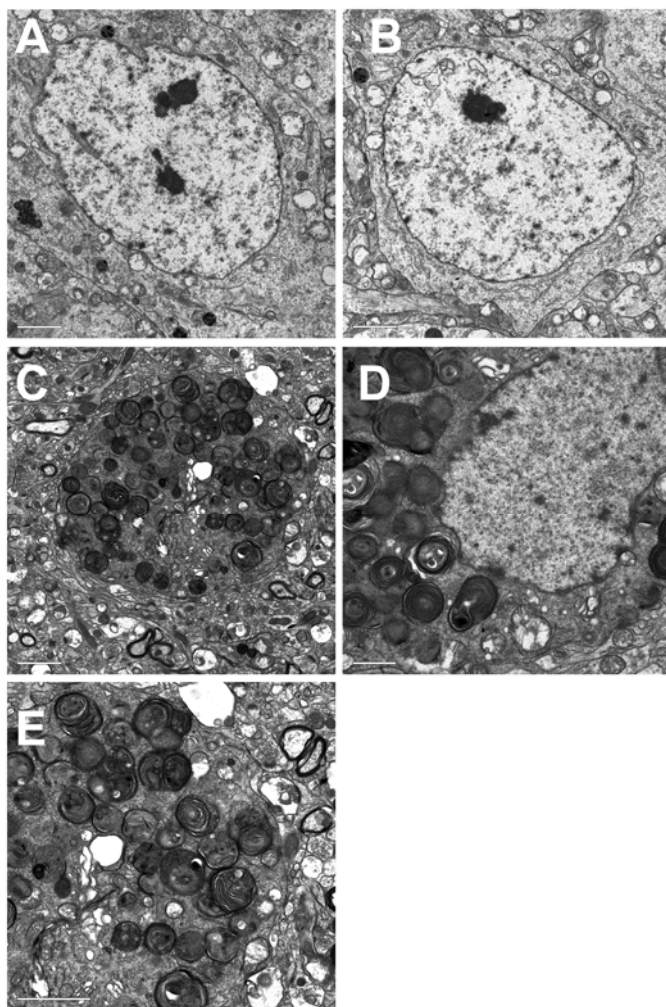

Supplement: Figure S10 — Electron micrographs of hippocampal neurons from wild type (A), single Neu4−/− (B) and Hexa−/− (C) knockouts, and double ( Neu4−/−;Hexa−/− ) knockouts without (D) and with seizures (E). Affected neurons containing vacuolated cytoplasm are only present in B, C and D. Bars range between 1 and 2 µm. The panels show representative images of at least 20 panels studied for 3 mice in each group. (0.34 MB PDF) [file pgen.1001118.s010.pdf]

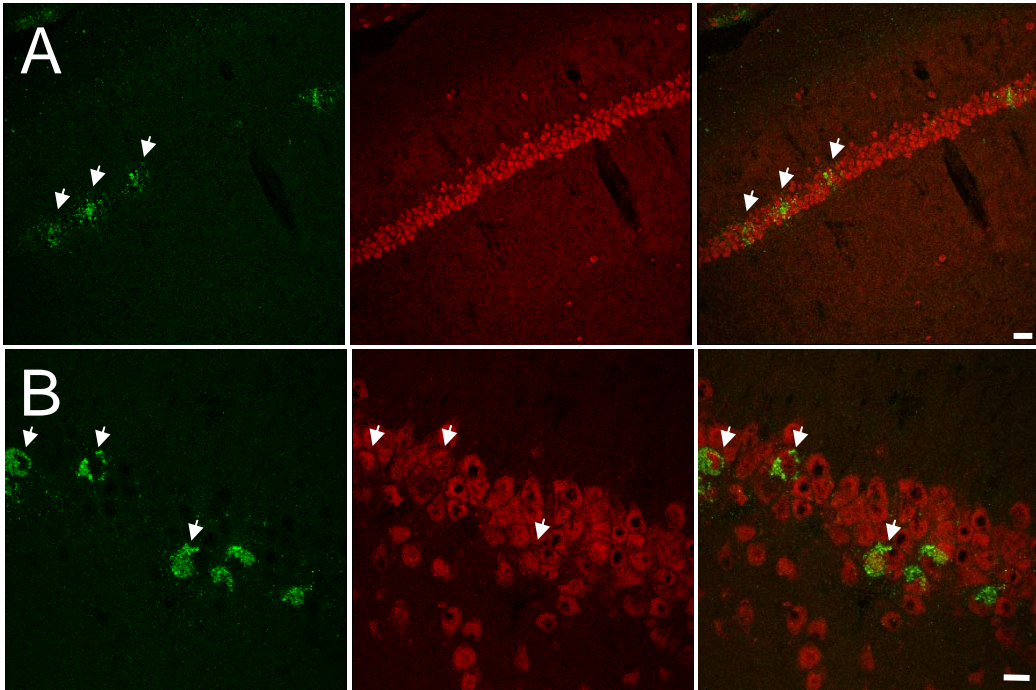

Supplement: Figure S11 — GM2 ganglioside is stored by sparse neurons in CA1 region of the hippocampus of Neu4−/−;Hexa−/− mouse with seizures. (A) GM2 ganglioside (green, right panels) and NeuN (red, middle panels) immunostaining in CA1 region of the hippocampus in a Neu4−/−;Hexa−/− mouse with seizures. NeuN staining is stronger in the perinuclear region of neuronal somata. (B) High magnification images showing that GM2 ganglioside is stored by neurons in the pyramidal layer (arrows). Scale bars: A, 50 µm; B, 20 µm. The panels show representative images of at least 15 panels studied for 3 mice in each group. (0.52 MB PDF) [file pgen.1001118.s011.pdf]

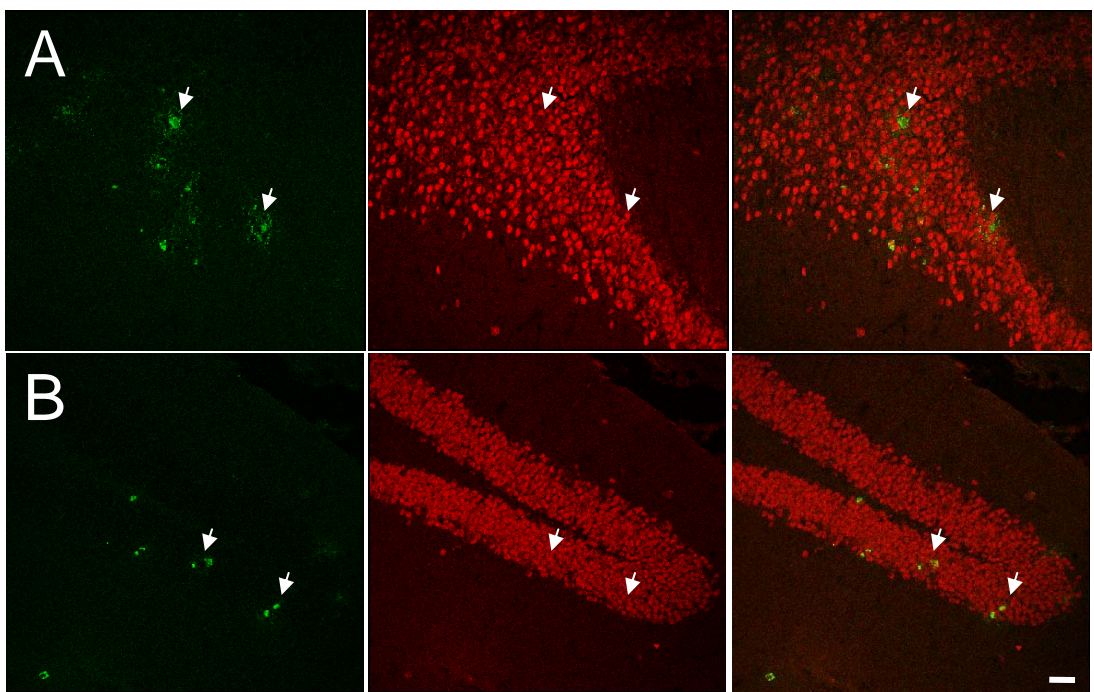

Supplement: Figure S12 — GM2 ganglioside is stored by sparse neurons in CA3 and dentate gyrus regions of the hippocampus of double KO, epileptic mouse. Panels show GM2 ganglioside (green, right panel) and NeuN (red, middle panels) immunostaining in CA3 (A) and dentate gyrus (B) regions of the hippocampus in a double Neu4−/−;Hexa−/− mouse with seizures. GM2 colocalizes with NeuN (arrows). Scale bar: 100 µm. Panels show representative images of at least 15 panels studied for 3 mice in each group. (0.54 MB PDF) [file pgen.1001118.s012.pdf]

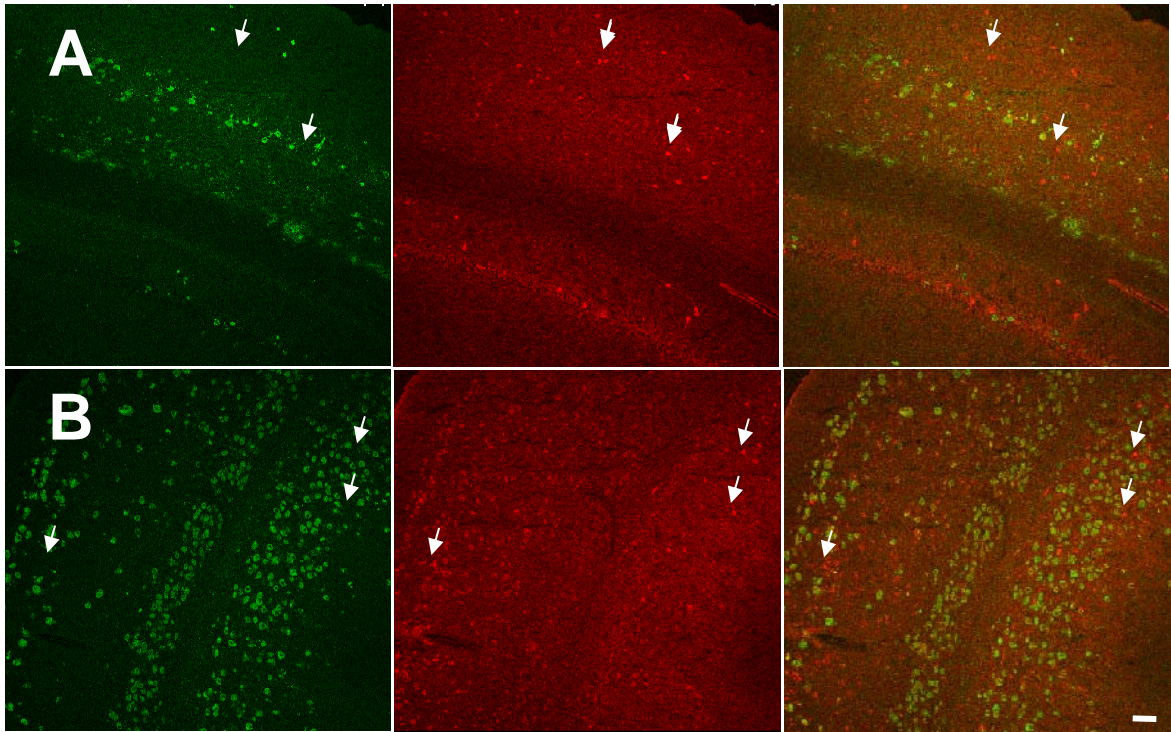

Supplement: Figure S13 — GM2 ganglioside is not stored by parvalbumin-positive GABAergic interneurons in cortex of Neu4−/−;Hexa−/− mouse with seizures. Panels show GM2 ganglioside (green, right panel) and NeuN (red, middle panels) immunostaining in dorsal (A) and ventral (B) cortex in a Neu4−/−;Hexa−/− mouse with seizures. Note that Parvalbumin-positive neurons do not store GM2 (arrows). Parvalbumin is expressed by about 50% of cortical GABAergic interneurons. Scale bar: 100 µm. (0.39 MB PDF) [file pgen.1001118.s013.pdf]

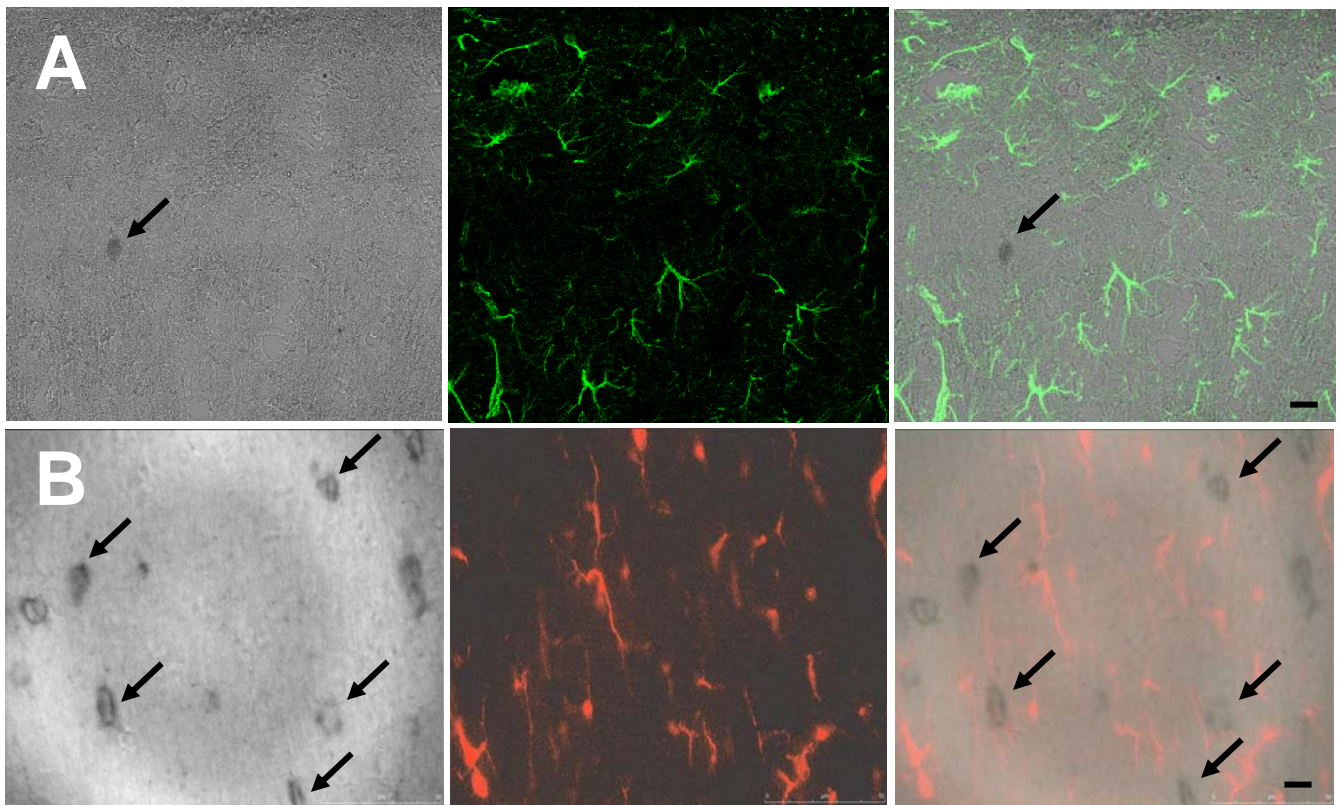

Supplement: Figure S14 — Neu4 is not expressed by astrocytes or microglia cells. In situ hybridization with a Neu4 probe labels few sparse cells in CA1 region of the hippocampus (arrows, left and right panels). These cells are negative for immunostaining for the astrocyte marker GFAP (A, green, middle panel) and microglial marker Eba1a (B, red, middle panel) Scale bar: 20 µm. The panels show representative images of at least 10 panels studied. (0.36 MB PDF) [file pgen.1001118.s014.pdf]
